# Validation of the Positive and Negative Affect Schedule (PANAS) in older Argentine adults

**DOI:** 10.1590/1980-5764-DN-2025-0441

**Published:** 2026-07-20

**Authors:** Natalia Irrazabal, Carolina Feldberg, Fernando Tonini, Silvia Deborah Ofman, Maria del Rosario Quian, Alejandro Castro Solano

**Affiliations:** 1 Universidad de Palermo, Buenos Aires, Argentina.; 2 Consejo Nacional de Investigaciones Científicas y Técnicas, Buenos Aires, Argentina.; 3 Instituto de Neurociencias Buenos Aires, Buenos Aires, Argentina.

**Keywords:** Affect, Aged, Health of the Elderly, Psychometrics, Validation Study, Afecto, Ancianos, Salud del Anciano, Psicometría, Estudio de Validación

## Abstract

Emotional functioning plays a key role in well-being and healthy ageing. The Positive and Negative Affect Schedule (PANAS) is one of the most widely used tools to assess affective dimensions, but few studies have validated its psychometric properties in older adults in Argentina.

**Objective:**

To validate the factorial structure and psychometric properties of the PANAS in a sample of non-institutionalised older Argentine adults.

**Methods:**

A total of 206 participants aged 60–91 years completed the PANAS along with measures of emotional, psychological, and social well-being, life satisfaction, and depression. Confirmatory factor analysis was performed to test the bifactorial structure of positive affect (PA) and negative affect (NA), and reliability was assessed using Cronbach’s α and McDonald’s ω.

**Results:**

The two-factor model showed excellent fit to the data (Comparative Fit Index=0.98; Root Mean Square Error of Approximation=0.06), supporting the distinction between PA and NA. Internal consistency was high for both dimensions (α and ω>0.80). As expected, PA correlated positively with emotional and personal well-being and life satisfaction, whereas NA correlated negatively with these variables and positively with depressive symptoms.

**Conclusion:**

The findings confirm the validity and reliability of the PANAS in older Argentine adults. This instrument demonstrates a robust bidimensional structure and consistent associations with relevant psychological constructs, supporting its use for assessing affective dimensions in research and clinical contexts involving ageing populations.

## RESUMEN

El funcionamiento emocional cumple un papel central en el bienestar y el envejecimiento saludable. La Escala de Afecto Positivo y Negativo (PANAS) es una de las herramientas más utilizadas para evaluar las dimensiones afectivas; sin embargo, existen pocos estudios que hayan validado sus propiedades psicométricas en adultos mayores argentinos.

**Objetivo:**

Validar la estructura factorial y las propiedades psicométricas de la PANAS en una muestra de adultos mayores argentinos no institucionalizados.

**Métodos:**

Participaron 206 adultos mayores entre 60 y 91 años, quienes completaron la PANAS junto con medidas de bienestar emocional, psicológico y social, satisfacción con la vida y depresión. Se realizó un análisis factorial confirmatorio para examinar la estructura bifactorial de afecto positivo (AP) y afecto negativo (AN), y se evaluó la confiabilidad mediante los coeficientes alfa de Cronbach y omega de McDonald.

**Resultados:**

El modelo de dos factores mostró un excelente ajuste a los datos (Comparative Fit Index=0,98; Root Mean Square Error of Approximation=0,06), confirmando la distinción entre AP y AN. La consistencia interna fue alta para ambas dimensiones (α y ω>0,80). El AP se asoció positivamente con el bienestar emocional y personal, así como con la satisfacción con la vida, mientras que el AN se relacionó negativamente con estas variables y positivamente con los síntomas depresivos.

**Conclusión:**

Los resultados confirman la validez y confiabilidad de la PANAS en adultos mayores argentinos, respaldando su estructura bidimensional y su utilidad para evaluar las dimensiones afectivas en contextos clínicos y comunitarios.

## INTRODUCTION

Given the steady rise in the older adult population in recent decades, there is an increasing need to examine the factors that promote health and well-being in this demographic group^1^. Healthy ageing is a multifaceted process influenced by genetic, behavioural, and psychological factors^2^. Among these, emotional functioning plays a central role, as it is strongly linked to health, social relationships, and overall quality of life in later life^3^. Maintaining a high level of subjective well-being is considered a key indicator of healthy ageing^4^. According to socioemotional selectivity theory, as people age, they increasingly prioritise emotionally meaningful goals and experiences, leading to greater positive affect and lower negative affect^5^. Moreover, emotion regulation capacities are often preserved or even enhanced in late adulthood, supporting resilience and psychological well-being despite age-related challenges^6^. Understanding the interplay between ageing and emotional functioning is therefore essential for promoting mental health and adaptive coping in this population.

Positive affect (PA) and negative affect (NA) have been widely used as broad dimensions to describe emotional experience and represent the affective components of subjective well-being^7,8^. To study these dimensions, valid and culturally appropriate measures are necessary. However, there is a scarcity of instruments specifically validated for older adults.

The dimensional model of emotion, initially proposed by Wundt^9^ and later developed by Watson and Tellegen^10^ and Russell and Barrett^11^, conceptualises emotions within a two-dimensional space defined by valence (pleasant–unpleasant) and arousal (high-low). This framework allows researchers to capture the full range of emotional experiences without resorting to discrete emotion categories. Within this perspective, affect has been operationalised mainly through two orthogonal dimensions — PA and NA^10,12^. PA reflects high energy, concentration, and pleasurable engagement, while NA involves distress and aversive states such as fear, guilt, or irritability^13^. Functionally, PA is linked to the activation of the approach system, promoting exploration, creativity, and social cooperation, whereas NA activates avoidance responses, helping individuals manage threats and regulate emotions in adverse contexts^14^.

The Positive and Negative Affect Schedule (PANAS)^13^ is among the most widely used and validated measures of affect. It consists of 20 adjectives (10 for PA, 10 for NA) and can assess either state or trait affect depending on the temporal instructions. Psychometric analyses have shown high internal consistency (α=0.86–0.90 for PA; α=0.84–0.87 for NA) and low intercorrelation between the two dimensions (*r*=-0.12 to -0.23), supporting discriminant validity. Test–retest studies further indicate adequate temporal stability. In terms of external validity, PA and NA show distinct correlations with anxiety, depression, and distress as measured by instruments such as the Hopkins Symptom Checklist (HSCL)^15^, the Beck Depression Inventory (BDI)^16^, and the State-Trait Anxiety Inventory (STAI)^17^, reinforcing the PANAS’s clinical relevance.

The PANAS has been extensively adapted across cultures, demonstrating its robustness. In Spain, Sandín et al.^18^ confirmed the bifactorial structure with high internal consistency (α>0.87) and independence between PA and NA. Later studies^19^ replicated these results in both clinical and non-clinical samples, showing expected associations with well-being and psychological distress. Similar findings were reported in Mexico^20^, Italy^21^, and Canada^22^. Some studies suggested a three-factor model dividing NA into anxiety- and anger-related subfactors^22,23^, but the two-factor model remains the most consistent across contexts.

In Argentina, several studies have validated the PANAS across different populations, mainly in Córdoba. Schulz-Begle et al.^24^ adapted the instrument for children aged 8–11, confirming a bifactorial structure and adequate reliability, though recommending replication with broader samples. Moriondo et al.^14^ validated the scale in a general adult population, confirming two factors (α=0.82 for PA; α=0.83 for NA) and their independence, while emphasising the role of cultural interpretation in item responses. Medrano et al.^25^ replicated the structure in university students and found that older adults scored higher on both PA and NA, possibly reflecting greater emotional awareness. Flores Kanter and Medrano^26^ compared bifactorial and trifactorial models in athletes, concluding that both were adequate but that distinguishing anxiety- and anger-related NA improved model precision — an important differentiation in competitive settings. Finally, Caicedo-Cavagnis et al.^27^ validated the PANAS in general population, students, and athletes, testing seven factorial models. They found that removing two items (“alert” and “excited”) improved model fit while preserving reliability, supporting both two- and three-factor structures.

Since the development of the PANAS, several adaptations have examined its performance in older adults^28,29,30,31,32^. Kercher^33^ validated a shortened 10-item version in U.S. older adults, confirming the original structure but noting cultural and educational differences that affected item comprehension. Similarly, Hilleras et al.^29^ replicated the two-factor model in Swedish adults aged 90 and above. Other studies used versions with different item counts to address population-specific needs. For example, Ready et al.^32^ used an extended form and found that, although the bifactorial structure held, some items were harder for older adults to interpret. In Spanish-speaking contexts, Buz et al.^28^ analysed the full PANAS in older adults in Spain, confirming two orthogonal factors with good psychometric properties and factorial invariance across age and sex. This finding supports the scale’s stability within older populations. von Humboldt et al.^8^ examined its cross-cultural robustness in participants from Portugal, Angola, Brazil, and the United Kingdom, noting that correlations between PA and NA varied slightly by culture. More recently, Lemos et al.^30^ validated a condensed 14-item PANAS among institutionalised older adults in Portugal, confirming a clear two-factor structure with satisfactory reliability, convergent/divergent validity, and temporal stability. Their findings underscore the PANAS’s utility as a concise and reliable instrument for assessing affect in institutional settings.

Taken together, prior studies confirm the PANAS as a reliable and valid instrument for assessing affective dimensions across different populations and cultures. In Argentina, the existing evidence supports the two-factor structure and sound psychometric properties. However, continued refinement through confirmatory factor analyses and cross-age or cultural comparisons remains essential.

Despite the growing body of validation research, psychometric studies focused specifically on older adults in Latin America remain scarce, particularly in Argentina. This represents a significant gap in current knowledge. Older adults possess distinct cognitive, emotional, and cultural characteristics that may influence how they understand and express affect, making it crucial to validate appropriate tools for this group. Considering the rapid growth of the ageing population and the increasing interest in promoting psychological well-being in later life, the availability of validated and culturally adapted instruments is essential. Although several Argentine adaptations of the PANAS exist for general or mixed-age populations, no study has specifically validated the instrument in non-institutionalised older Argentine adults. This absence limits the availability of local psychometric data on factorial structure, internal consistency, and convergent/divergent validity in this demographic. Establishing such evidence is important for improving affective assessment, developing clinical interventions, and guiding emotional health promotion and public policy initiatives for healthy ageing.

Accordingly, the present study aimed to validate the factorial structure and assess the psychometric properties of the PANAS in a sample of non-institutionalised older Argentine adults. It was hypothesised that the instrument would exhibit a bidimensional structure, good reliability, and expected correlations with measures of well-being and depression.

## METHODS

### Participants

The sample comprised 206 older adults from the general population of Buenos Aires, Argentina. Inclusion criteria were age 60 or older and non-institutionalised status; the exclusion criterion was a Montreal Cognitive Assessment (MoCA) score below 22. The mean age was 73.40 years (SD=7.35, range=60–91), with 75.2% women and 24.8% men. Initially, 232 individuals participated, but 26 were excluded due to incomplete data or cognitive impairment. The screening ensured that all participants exhibited adequate cognitive functioning to maintain data validity. Regarding education, 31.6% completed secondary school, and 68.4% reported full or partial university studies.

### Instruments

#### Mental Health Continuum-Short Form (MHC–SF^34^; Argentine adaptation^35^)

This 14-item measure assesses emotional, social, and psychological well-being through a 6-point Likert scale (0=never to 5=every day). The Argentine version confirmed a three-factor model and invariance across sex and age. In the current sample, exploratory structural equation modelling showed good fit (Comparative Fit Index [CFI]=0.97, Tucker-Lewis Index [TLI]=0.97, Root Mean Square Error of Approximation [RMSEA]=0.047 [0.03–0.08]). Internal consistency was satisfactory: emotional well-being α=0.73 [ω=0.74], psychological well-being α=0.78 [ω=0.78], social well-being α=0.72 [ω=0.74], and overall α=0.83 [ω=0.85].

#### Satisfaction with Life Scale (SWLS**7**; Argentine adaptation^36^)

The SWLS contains five items evaluating the cognitive component of well-being. Respondents indicate agreement on a 7-point Likert scale. Confirmatory factor analysis indicated excellent fit (CFI=0.99, TLI=0.98, Standardized Root Mean Square Residual [SRMR]=0.01, RMSEA=0.050 [0.03–0.07]). Reliability was high (α=0.86; ω=0.87).

#### Geriatric Depression Scale (GDS^37^; Argentine adaptation^38^)

This 15-item dichotomous scale (0=No, 1=Yes) assesses depressive symptoms experienced during the past week. It is widely used in community and clinical contexts with older adults. Previous research has shown adequate fit (CFI=0.97, Normed Fit Index [NFI]=0.95, RMSEA=0.060 [0.05–0.07]). In the present study, internal consistency was acceptable (Kuder-Richardson Formula 20 [KR-20]=0.70; ω=0.75).

#### Montreal Cognitive Assessment (MoCA^39,40^; Argentine validation^40^)

The MoCA screens global cognition across multiple domains, including memory, executive function, language, and visuospatial ability. It requires approximately 10 minutes to administer, with a maximum score of 30. Scores below 26 suggest possible cognitive impairment. The measure shows high internal consistency (α=0.88) and good test–retest reliability.

#### Positive and Negative Affect Schedule (PANAS^13^; Argentine version^27^)

The Argentine adaptation includes 18 items: 8 for PA (e.g., interested, inspired) and 10 for NA (e.g., distressed, guilty). Participants rate how intensely they experienced each affect during the last days on a 5-point scale (1=very slightly or not at all to 5=extremely). Subscale scores are obtained by summing corresponding items. The present study aimed to validate this version for non-institutionalised older adults.

### Procedure

Data were collected in person during a single session at a science cultural centre in Buenos Aires. Administration was carried out in groups of approximately 25 participants and lasted between 30 and 40 minutes. All questionnaires were paper-based and supervised by trained researchers.

### Ethical considerations and data analysis

Participation was voluntary and non-remunerated. Informed consent was obtained prior to data collection, and confidentiality and anonymity were guaranteed. Participants were informed of their right to withdraw at any time without consequences. The study complied with the APA Ethical Principles (2017), NC3Rs guidelines, and CONICET standards (Resolution No. 2857/2006) and was approved by the INEBA Ethics Committee (Code: 11008).

Descriptive analyses and correlations were performed using Jamovi^41^. Confirmatory factor analyses were conducted with the lavaan package^42^ in R (version 4.3.2)^43^. Model fit was evaluated using standard indices (χ^2^, CFI, TLI, RMSEA, SRMR). Reliability was assessed via Cronbach’s α and McDonald’s ω coefficients. The data and accompanying data dictionary used for the analyses reported in this study are available at the following Open Science Framework (OSF) repository link: https://shorturl.at/lGtmD

## RESULTS

### Descriptive analysis

Table 1 presents the descriptive analyses for each item, as well as for the PA and NA scales of the PANAS. Additionally, the correlations between each item and the total score of its corresponding scale are reported.

**Table 1:** Descriptive statistics of the Positive and Negative Affect Schedule (PANAS) and item–total scale correlations.

	Mean	SD	r (item-total)
Interesado (interested)	3.76	0.88	0.46
Fuerte (strong)	3.49	0.98	0.64
Entusiasmado (enthusiastic)	3.75	0.97	0.70
Orgulloso (proud)	3.13	1.15	0.61
Inspirado (inspired)	3.28	1.15	0.67
Decidido (determined)	3.61	1.00	0.74
Atento (attentive)	3.83	0.99	0.73
Activo (active)	4.10	0.92	0.71
PANAS (positive)	3.60	0.70	
Afligido (distressed)	2.18	1.15	0.66
Disgustado (upset)	1.85	1.05	0.68
Culpable (guilty)	1.33	0.74	0.59
Asustado (scared)	1.59	0.86	0.64
Hostil (hostile)	1.34	0.73	0.58
Irritable (irritable)	1.64	0.84	0.58
Avergonzado (ashamed)	1.18	0.59	0.39
Nervioso (nervous)	2.14	1.03	0.69
Intranquilo (jittery)	2.27	1.11	0.74
Temeroso (afraid)	1.70	0.91	0.72
PANAS (negative)	1.72	0.58	

Abbreviation: SD, standard deviation; r, correlation.

### Assessment of response bias and data quality

To evaluate data quality and the potential presence of response style–related biases, complementary analyses were conducted to detect both mechanical responding patterns and atypical multivariate response profiles. First, the *longstring* index was calculated, defined as the maximum number of identical consecutive responses across the administered items. In the total sample (n=206), the index showed a mean of 4.32 (standard deviation [SD]±2.10), a median of 4, a minimum value of 2, and a maximum value of 14. The 90th percentile was 6 and the 95th percentile was 7, indicating that the vast majority of participants exhibited short sequences of identical responses. Only 4.4% of the sample showed values equal to or greater than 8, a conservative threshold commonly used to identify potential mechanical responding. Taken together, these findings suggest adequate data quality and the absence of systematic automated response styles in the analyzed sample.

Complementarily, the presence of atypical multivariate response profiles was examined using Mahalanobis distance based on the PANAS items. A conservative criterion (p<0.001) was adopted, classifying cases exceeding the critical value of the χ^2^ distribution with 18 degrees of freedom as multivariate outliers. Results indicated that only 3.9% of the sample (n=8) met this criterion. Replication of the confirmatory factor analysis excluding these cases did not reveal substantive changes in model fit indices or factor loadings; therefore, the full sample was retained for the final analyses.

### Reliability

Cronbach’s α coefficients obtained for the scales were 0.81 for PA and 0.82 for NA. Given that PANAS items are measured on an ordinal Likert scale, McDonald’s ω coefficient, which is more appropriate for this type of item, was also calculated. The results were ω=0.82 for the PA scale and ω=0.83 for the NA scale, indicating adequate internal consistency for both dimensions.

To assess item homogeneity, item–total scale correlations were calculated for each dimension. For the PA scale, the correlations ranged from 0.46–0.71, whereas for the NA scale, they ranged from 0.59–0.74. These values indicate adequate internal consistency and suggest that the items significantly contribute to measuring their respective constructs.

### Internal structure analysis

A confirmatory factor analysis (CFA) using structural equation modelling (SEM) was conducted to assess the bidimensional structure of the PANAS. In this model, PA items were specified to load onto a single factor, and NA items were specified to load onto another. Since the items were rated on an ordinal scale, the weighted least squares mean and variance adjusted (WLSMV) estimation method was employed, as recommended for ordinal data. The model results indicated a good fit to the empirical data, as reflected in the fit indices: CFI=0.98, TLI=0.97, SRMR=0.08, RMSEA=0.06 [90% confidence interval [CI] 0.04–0.07]. These values support the bidimensional structure of the instrument. Standardized factor loadings for the PA latent variable ranged from 0.51–0.76, and those for NA ranged from 0.50–0.82. These results indicate moderate associations between the items and their respective latent constructs, meeting the acceptable standards for this type of analysis^44^.

### Concurrent validity

Hypotheses were formulated regarding the associations between the PANAS scales and other related measures. Negative associations were expected between the NA scale and the GDS, as were positive associations between the NA scale and the PA scale. Additionally, positive associations were hypothesized between the PA scale and the dimensions of emotional, psychological, and social well-being, along with negative associations between PA and NA. It was also hypothesized that PA would be positively associated with life satisfaction, whereas NA would have a negative relationship with life satisfaction. As shown in Table 2, Pearson correlations supported the hypothesized associations, except for the relationship between social well-being and NA. The correlations ranged from moderate to large in effect size^45^.

**Table 2:** Correlations between positive affect, negative affect, depression, emotional, psychological, and social well-being, and life satisfaction (n=206).

	Positive affect	p-value	Negative affect	p-value
Depression	0.23	0.001	-0.34	0.001
Emotional well-being	0.35	0.001	-0.36	0.001
Personal well-being	0.28	0.001	-0.25	0.001
Social well-being	0.15	0.030	-0.12	0.070
Satisfaction with life	0.31	0.001	-0.29	0.001

## DISCUSSION

This study aimed to validate the factorial structure and psychometric properties of the PANAS scale in a sample of non-institutionalised older Argentine adults. The findings lend support to the original bifactorial structure proposed by Watson et al.^13^, with a satisfactory model fit to the empirical data, acceptable internal consistency for both dimensions, and convergent correlations with other instruments that provide evidence in support of its concurrent validity.

The confirmatory factor analysis indices are consistent with those reported in previous studies of Argentine populations (e.g.,^14,27^) and older adults from other countries (e.g.,^28,30^). This finding serves to substantiate the stability of the bidimensional model in this particular age group. The obtained reliability coefficients were satisfactory (α and ω>0.80 for both scales) and comparable to standards reported in earlier studies, suggesting that the PANAS retains its internal robustness even in older adults. Furthermore, item-total scale correlations confirmed the internal homogeneity of the items, with no evidence of problematic or weak items.

With regard to concurrent validity, the results confirmed the expected associations between PANAS dimensions and measures of well-being and psychological distress. The present study found a significant positive correlation between positive psychological adjustment and emotional well-being, psychological well-being, and life satisfaction. These findings are consistent with the extant empirical literature on psychological well-being^7,34^. In contrast, NA demonstrated negative correlations with these variables and a positive relationship with the GDS. This finding is consistent with studies associating negative affectivity with elevated levels of depressive symptoms and subjective distress^13,28^.

It is noteworthy that while all the observed correlations were statistically significant and demonstrated moderate to large effect sizes, the association between NA and social well-being was only marginally significant. This phenomenon could be ascribed to characteristics of the social well-being construct in late life, or alternatively, to limitations in the instrument’s capacity to discern aspects pertaining to social integration. This issue must be given due consideration in future studies.

In addition to their relevance for subjective well-being, affective changes in later life have been increasingly linked to cognitive functioning and everyday functionality. Emotional states such as reduced PA or elevated NA have been associated with poorer cognitive performance, increased cognitive complaints, and declines in instrumental activities of daily living, even in older adults without a formal diagnosis of dementia. From this perspective, affective functioning constitutes an important dimension of mental health that may interact with cognitive and functional domains during ageing.

Mild behavioral impairment^46^ has been proposed as a late-life neurobehavioral syndrome characterised by the emergence and persistence of neuropsychiatric symptoms in cognitively normal individuals or those with mild cognitive impairment, and has been associated with an increased risk of subsequent cognitive decline and dementia. These symptoms include affective dysregulation, apathy, anxiety, irritability, and mood changes emerging in cognitively preserved older adults. Importantly, mild behavioral impairment has been suggested as a potential prodromal manifestation of neurodegenerative processes and as a risk marker for future cognitive decline and dementia^47,48^.

Although the present study did not assess cognitive trajectories, functional outcomes, or longitudinal progression, the affective dimensions captured by the PANAS may be conceptually relevant within this framework. Elevated NA or diminished PA, when observed in cognitively intact older adults, could represent early behavioral or emotional changes that merit closer clinical attention. In this sense, the PANAS could contribute to the identification of affective risk profiles that, while not diagnostic, may signal vulnerability to future cognitive or functional deterioration. It is important to emphasise that the PANAS is not intended as a screening or diagnostic tool for dementia or prodromal cognitive disorders. Rather, its potential value lies in complementing cognitive and functional assessments by providing systematic information on emotional functioning. Future longitudinal studies integrating affective, cognitive, and functional measures are needed to determine whether changes detected by the PANAS are associated with progression toward mild cognitive impairment or dementia. Such research would help clarify the role of affective symptoms as early indicators of neurodegenerative risk in ageing populations.

The findings provide empirical evidence for the relevance of the PANAS as a valid and reliable instrument for assessing PA and NA in older Argentine adults. Its application can be particularly useful in clinical, community, or research contexts, where the identification of affective profiles contributes to a comprehensive approach to assessing psychological well-being in ageing individuals. Furthermore, the validation of these findings in a non-institutionalised population provides novel evidence in this field, as most prior studies have focused on institutionalised older adults or on mixed samples (e.g.,^30^). These findings underscore the importance of using culturally validated affective measures in clinical gerontology and policy design for ageing populations.

However, it is important to acknowledge the limitations of this study. Firstly, the sample was composed predominantly of women and individuals with relatively high educational levels, which may limit the generalizability of the results to the broader population of older Argentine adults. Secondly, the cross-sectional design of the study prevents the assessment of the instrument’s temporal stability and limits causal inferences regarding the directionality of the observed relationships between affectivity and well-being or distress variables. Longitudinal designs would allow for the evaluation of test–retest reliability and the examination of potential changes in affective dimensions over time, as well as their relationship with cognitive, functional, and mental health outcomes in later life. Thirdly, factorial invariance analyses were not conducted due to sample size constraints and demographic imbalances, such as sex, advanced age, and educational level, which would be relevant for confirming the metric equivalence of the instrument across different subgroups. It is recommended that subsequent research examine factorial invariance as a function of sociodemographic variables. This would assist in strengthening the psychometric foundation of the instrument within the specified population. Finally, the present study did not include explicit control for medical comorbidities. In geriatric populations, chronic physical conditions, pain, functional limitations, and polypharmacy may overlap with affective experiences and influence responses to both PA and NA items. Physical illness burden, for example, may artificially inflate NA scores or attenuate PA, posing a potential challenge to discriminant validity. Future studies would benefit from incorporating standardized indices of medical comorbidity, such as the Charlson Comorbidity Index, as covariates in psychometric or structural models. This approach would allow for a clearer estimation of latent affective constructs while accounting for variance associated with physical health status. Although this limitation does not invalidate the present findings, it highlights an important methodological consideration for refining affective assessment in older adults.

In conclusion, the present study provides robust evidence supporting the validity, reliability, and feasibility of the PANAS for use in non-institutionalised older Argentine adults. The confirmatory factor analysis confirmed a stable bidimensional structure of PA and NA, with satisfactory internal consistency for both dimensions, comparable to those reported in international and local studies.

Beyond its psychometric robustness, the PANAS proved to be a brief, easily administrable, and well-accepted instrument among older adults, supporting its feasibility for use in both research and applied settings. Its concise format and clear affective descriptors make it particularly suitable for community, clinical, and epidemiological contexts involving ageing populations.

The consistent associations observed between affective dimensions, well-being indicators, life satisfaction, and depressive symptoms further reinforce the construct validity of the instrument. Taken together, these findings support the PANAS as a valuable tool for assessing emotional functioning in older adults and for contributing to a comprehensive evaluation of psychological well-being in later life. Its use may inform preventive strategies, clinical screening, and the design of interventions aimed at promoting healthy ageing and emotional well-being in the Argentine context.

## Data Availability

The datasets generated and/or analysed during the current study are available at the Open Science Framework OSF repository (https://shorturl.at/lGtmD).
